# Lysergic acid diethylamide stimulates cardiac human H_2_ histamine and cardiac human 5-HT_4_-serotonin receptors

**DOI:** 10.1007/s00210-023-02591-6

**Published:** 2023-07-04

**Authors:** Ulrich Gergs, Hannes Jacob, Pauline Braekow, Britt Hofmann, Steffen Pockes, Laura J. Humphrys, Uwe Kirchhefer, Charlotte Fehse, Joachim Neumann

**Affiliations:** 1 Institute for Pharmacology and Toxicology, Medical Faculty, Martin Luther University Halle-Wittenberg, Magdeburger Straße 4, 06097 Halle (Saale), Germany; 2 Department of Cardiac Surgery, Mid-German Heart Center, University Hospital Halle, Ernst Grube Straße 40, 06097 Halle (Saale), Germany; 3 Institute of Pharmacy, University of Regensburg, Universitätsstraße 31, 93040 Regensburg, Germany; 4 Institute for Pharmacology and Toxicology, University Hospital Münster, Westfälische Wilhelms-University, Domagkstraße 12, 48149 Münster, Germany

**Keywords:** Lysergic acid diethylamide, H_2_-histamine receptor, Heart, Inotropy, Chronotropy

## Abstract

Lysergic acid diethylamide (LSD) is an artificial hallucinogenic drug. Thus, we hypothesized that LSD might act 5-HT_4_ serotonin receptors and/or H_2_ histamine receptors. We studied isolated electrically stimulated left atrial preparations, spontaneously beating right atrial preparations, and spontaneously beating Langendorff-perfused hearts from transgenic mice with cardiomyocyte-specific overexpression of the human 5-HT_4_ receptor (5-HT_4_-TG) or of the H_2_-histamine receptor (H_2_-TG). For comparison, we used wild type littermate mice (WT). Finally, we measured isometric force of contraction in isolated electrically stimulated muscle strips from the human right atrium obtained from patients during bypass surgery. LSD (up to 10 µM) concentration dependently increased force of contraction and beating rate in left or right atrial preparations from 5-HT_4_-TG (*n* = 6, *p* < 0.05) in 5-HT_4_-TG atrial preparations. The inotropic and chronotropic effects of LSD were antagonized by 10 µM tropisetron in 5-HT_4_-TG. In contrast, LSD (10 µM) increased force of contraction and beating rate in left or right atrial preparations, from H_2_-TG. After pre-stimulation with cilostamide (1 µM), LSD (10 µM) increased force of contraction in human atrial preparations (*n* = 6, *p* < 0.05). The contractile effects of LSD in human atrial preparations could be antagonized by 10 µM cimetidine and 1 µM GR 125487. LSD leads to H_2_-histamine receptor and 5-HT_4_-receptor mediated cardiac effects in humans.

## Introduction

**Fig. 1:**
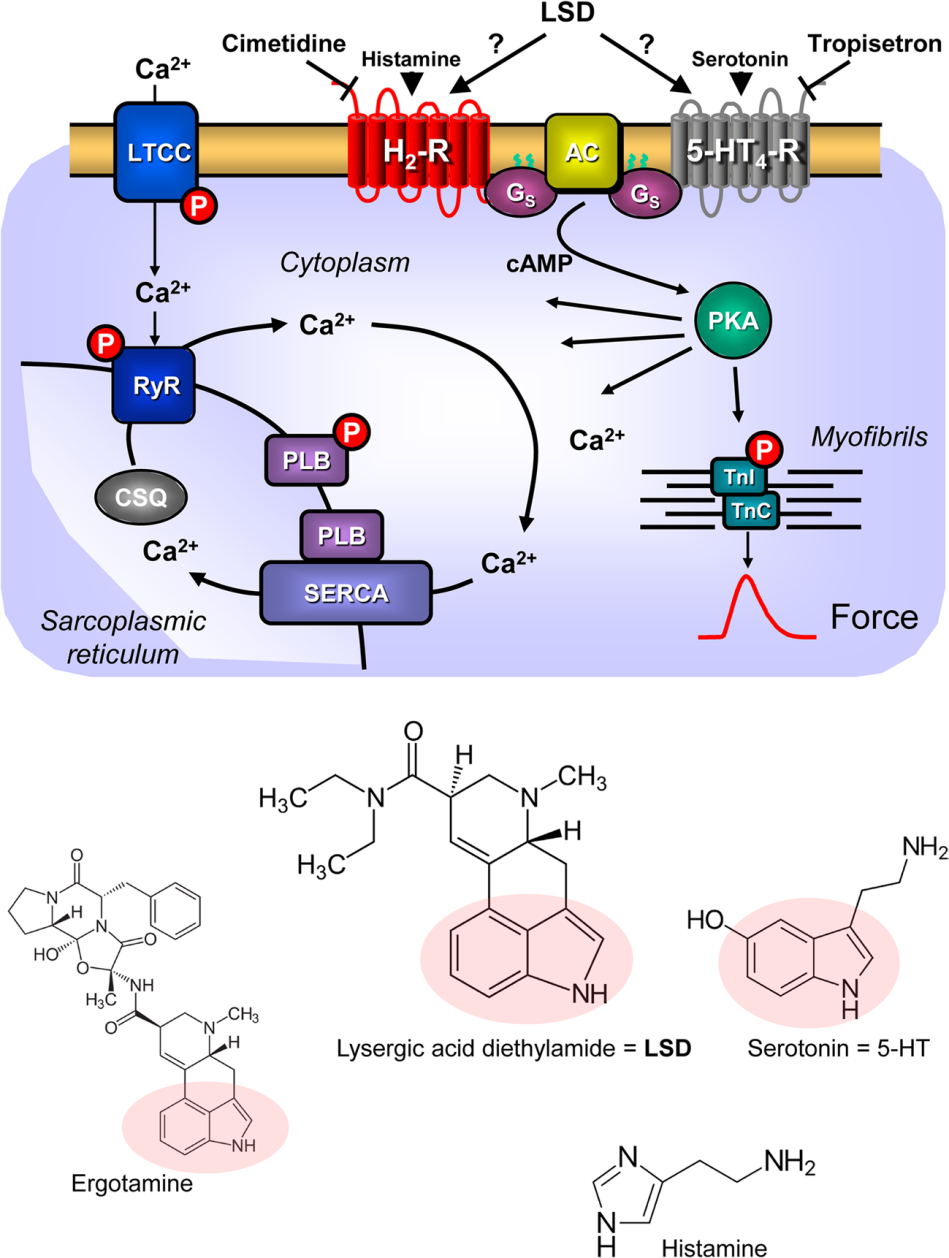
Top: Hypothetical action of lysergic acid diethylamide (LSD). LSD might activate 5-HT_4_-serotonin receptors (5-HT_4_-R, stimulated by serotonin and inhibited by tropisetron) or H_2_-histamine receptors (H_2_-R, stimulated by histamine and blocked by cimetidine) in the sarcolemma of cardiomyocytes. These receptor stimulations will converge into an activation of adenylyl cyclase activity (AC) by means of stimulatory guanosine triphosphate-binding proteins (G-proteins). AC produces 3´,5´-cyclic adenosine monophosphate (cAMP). This cAMP can then activate a cAMP-dependent protein kinase (PKA). This leads to phosphorylation of many target proteins (in red). For instance, the L-type Ca^2+^ channel (LTCC) is phosphorylated. This leads to enhanced entrance of trigger Ca^2+^ into the cell. Ca^2+^ can release Ca^2+^ from the sarcoplasmic reticulum. This Ca^2+^ can bind to myofilaments to generate force (red curve). In diastole, Ca^2+^ is removed from the cytosol. This leads to relaxation. Ca^2+^ is pumped by the enzyme SERCA into the sarcoplasmic reticulum where it binds to calsequestrin (CSQ). Ca^2+^ leaves the sarcoplasmic reticulum via the ryanodine receptor (RYR). Dephosphorylated phospholamban (PLB) inhibits SERCA. Phosphorylated PLB ceases to inhibit SERCA and thus Ca^2+^ is removed faster from the cytosol. In this way phosphorylation of PLB leads to faster relaxation. Relaxation is further augmented when PKA phosphorylates the troponin inhibitor (TnI) in the myofilaments containing troponin c (TnC). Bottom: Structural formulae of relevant molecules in the present study

Lysergic acid diethylamide (LSD, Fig. 1) was studied for use in psychiatry in the 1960s but was largely used in illicit ways and therefore removed from the market worldwide (review: Schlag et al. 2022). Currently, LSD is predominantly used for recreational purposes, and intoxications are recorded (review: Schlag et al. 2022).

Histamine acts via histamine H_1_, H_2_, H_3_ and H_4_ receptors (Panula et al. 2015; Neumann et al. 2021a, b, c). In the heart of mammals, all four histamine receptor subtypes have been described at RNA and/or protein level.

However, species differences exist in the cardiac effects of histamine (Neumann et al. 2021a, b, c). In the mouse, rat, dog and cat heart, a direct histamine receptor mediated inotropic or chronotropic effect is missing: inotropic effects of histamine were found to be indirect via release of endogenous catecholamines (Flacke et al. 1967; Dai et al. 1976; Laher and McNeill 1980a, 1980b, Gergs et al. 2019).

Moreover, even regional differences in the actions of histamine in the mammalian heart are known: in the rabbit atrium, H_1_ receptors are more prevalent and a positive inotropic effect mediated by H_1_ and phospholipase C activation has been described (Hattori et al. 1988). In contrast, in the rabbit ventricle a positive inotropic effect by H_2_ receptor activation via activation of adenylyl cyclase, a subsequent increase in cAMP and elevation of the activity of a cAMP dependent protein kinase (PKA) was noted by the same group (Hattori et al. 1990, 1991). In humans, H_2_ receptors were measurable in both the atrium and ventricle (radioligand binding: Baumann et al. 1982, 1983, 1984, antibody and RNA expression: Matsuda et al. 2004). In humans, the cardiac H_2_ receptors mediate a positive inotropic effect in isolated human atrial cardiac preparations (Levi et al., 1981, Genovese et al. 1988; Zerkowski et al. 1993; Thoren et al. 2011; Sanders et al. 1996). Infusion of histamine in patients led to an increase in heart beat and an increase in the first derivative of pressure development in the left ventricle (Vigorito et al. 1983). These effects of histamine in the human heart are not due to a release of noradrenaline: the human H_2_ receptor mediates the cardiac actions of histamine in isolated human cardiomyocytes in vitro where a release of noradrenaline from nerve cells was excluded (Sanders et al. 1996). An H_2_R- agonist called impromidine, has been shown to increase force of contraction in human cardiac preparations (Baumann et al. 1981, 1982, 1983).

LSD was classified by others as a partial agonist at rabbit and guinea-pig cardiac H_2_ receptors: at low concentrations LSD increases beating rate, and at high concentrations decreases beating rate, in isolated right atrial preparations from rabbits in a cimetidine sensitive fashion (Angus and Black 1980). Moreover, LSD antagonized the positive inotropic effect of histamine in isolated guinea pig papillary muscles (Angus and Black 1980). To the best of our knowledge, the effects of LSD on human cardiac H_2_ receptors have not been reported.

LSD can also act as an agonist at serotonin receptors. 5-HT_2A_ receptors mediate the hallucinogenic effects of LSD (Schlag et al. 2022). In the human heart, all inotropic and chronotropic effects of serotonin (5-HT) are 5-HT_4_ receptor mediated (reviews: Neumann et al 2017, 2023). We generated and characterized a mouse model with cardiomyocyte-specific overexpression of the human 5-HT_4_ receptor (5-HT_4_-TG) to investigate the actions of the receptor better (Gergs et al. 2010). In a similar fashion as described above for histamine, 5-HT in the heart of wild type littermate mice (WT) has no receptor mediated inotropic effects. However, in 5-HT_4_-TG, 5-HT exerted positive inotropic effects both in vivo and in vitro (Gergs et al. 2010, 2013). Based on this work from our group, we decided to use 5-HT_4_-TG to study a putative cardiac role of LSD via 5-HT_4_-receptors (Fig. 1). To test the clinical relevance of our findings, we set out to measure the effects of LSD under isometric conditions on the force of contraction in the human heart. To this end, we used electrically stimulated right atrial strips obtained rapidly from the surgical theatre.

In summary, we studied the following hypotheses: firstly, LSD stimulates contractility in H_2_-TG. Secondly, LSD stimulates contractility in 5-HT_4_-TG. Thirdly, LSD increases the force of contraction in the isolated human atrium via H_2_–histamine and/or 5-HT_4_–serotonin receptors.

## Methods

### Transgenic mice

The investigation conforms to the Guide for the Care and Use of Laboratory Animals published by the National Research Council (2011). Animals were maintained and handled according to approved protocols of the animal welfare committees of the University of Halle-Wittenberg, Germany. The generation and initial characterization of the transgenic mice has been described before (Gergs et al. 2010, 2019). In brief, for generation of transgenic mice by pronuclear DNA injection, human H_2_ -receptor cDNA or human 5-HT_4_ receptor cDNA were inserted into a mouse cardiac α-myosin heavy chain promoter expression cassette. For all experiments, adult transgenic mice and WT littermates of both sexes were used.

### Contractile studies in mice

As described before, the right or left atrial preparations from the mice were isolated and mounted in organ baths (Gergs et al. 2013; Neumann et al. 1998). The bathing solution of the organ baths contained 119.8 mM NaCI, 5.4 mM KCI, 1.8 mM CaCl_2_, 1.05 mM MgCl_2_, 0.42 mM NaH_2_PO_4_, 22.6 mM NaHCO_3_, 0.05 mM Na_2_EDTA, 0.28 mM ascorbic acid and 5.05 mM glucose. The solution was continuously gassed with 95% O_2_ and 5% CO_2_ and maintained at 37 °C and pH 7.4 (Neumann et al. 1998, Kim et al. 2004). Spontaneously beating right atrial preparations from mice were used to study any chronotropic effects. After equilibration was reached, ergometrine was cumulatively added to left atrial or right atrial preparations to establish concentration–response curves. Then, where indicated, either serotonin or histamine was additionally applied to the preparations. In separate experiments, concentration–response curves to ergotamine in mouse left atrial preparations were obtained and, after the effect of 10 µM ergotamine had reached a plateau, the atrial strips were rapidly brought to the temperature of liquid nitrogen for further study.

### Contractile studies on human preparations

The contractile studies on human preparations used the same setup and buffer as in the mouse studies. The samples were obtained from 3 male patients and 4 female patients, 78–82 years old. Drug therapy included β_1_-adrenoceptor antagonist metoprolol, the loop diuretic furosemide, the anticoagulant apixaban and the antithrombotic drug acetyl salicylic acid. Our methods used for atrial contraction studies in human samples have been previously published and were not altered in this study (Gergs et al. 2009, 2021b). Patients gave written informed consent.

### Langendorff-perfused hearts

Heart preparations were utilized as described previously (Kirchhefer et al. 2014; Gergs et al., 2020). Mice were anesthetized by intraperitoneally administered pentobarbital sodium (50 mg kg-1 body weight) and treated with 1.5 units of heparin. The hearts were removed from the opened chest, immediately attached by the aorta to a 20-gauge cannula, and perfused retrogradely under constant flow of 2 ml min^−1^ with oxygenized buffer solution (37 °C) containing (in mM): NaCI 119.8, KCI 5.4, CaCl_2_ 1.8, MgCl_2_ 1.05, NaH_2_P0_4_ 0.42, NaHCO_3_ 22.6, Na_2_EDTA 0.05, ascorbic acid 0.28 and glucose 5.0 in an in-house built isolated heart system equipped with a PowerLab system (ADInstruments, Oxford, United Kingdom). The heart preparations were allowed to equilibrate for 30 min before measurements. Hearts contracted spontaneously in sinus rhythm, and heart rate and force of contraction were measured and monitored continuously. The first derivative of left ventricular contraction (+ dF/dt and − dF/dt) was calculated (LabChart, ADInstruments, Oxford, United Kingdom). This was done to assess the rate of tension development and the rate of relaxation in order to measure the positive inotropic and lusitropic (relaxant) effects of the drugs we studied.

### Western blot analysis

Homogenates from ventricular tissue samples were prepared in 300 µl of 10 mM NaHCO_3_ and 100 µl 20% SDS. Crude extracts were incubated at 25 °C for 30 min before centrifugation to remove debris and thereafter, the supernatants (= homogenates) were separated and stored at -80 °C until further use. Western blot analysis was performed as previously described (Abella et al. 2023). Briefly, aliquots of 20 µg of protein were loaded per lane and finally, bands were detected using enhanced chemiluminescence (ECL, Amersham (Cytiva), Freiburg, Germany) together with an Amersham ImageQuant 800 imager (Cytiva, Freiburg, Germany). The following primary antibodies were used in this study: polyclonal rabbit anti phospho-PLB (antibody was raised against PLB-peptide phosphorylated at serine-16, Badrilla, Leeds, UK) and polyclonal rabbit anti calsequestrin (CSQ, abcam, Cambridge, UK). The characteristics and use of these antibodies has been reported repeatedly by our group (Kirchhefer et al., 2002). The antibody against calsequestrin was used as loading control.

### Radioligand competition binding

Radioligand competition binding experiments were performed as previously described by using the HEK293-SP-FLAG-hH2R cell line and [^3^H]DE257 (*K*_d_ = 66.9 nM, c = 40 nM) (Baumeister et al. 2015; Pockes et al. 2018; Rosier et al. 2021). Ligand dilutions were prepared tenfold concentrated in L-15 with 1% BSA, and 10 μL/ well was transferred to a flat-bottom polypropylene 96-well microtiter plate (Greiner Bio-One, Frickenhausen, Germany), as well as 10 μL/ well of the respective radioligand. The cells were adjusted to a density of 1.25 × 10^6^ cells/mL, and 80 μL of the cell suspension was added to each well (total volume of 100 μL). All data were analyzed using GraphPad Prism 9 software (San Diego, CA, USA). The normalized competition binding curves were then fitted with a four-parameter logistic fit yielding pIC_50_ values. These were transformed into p*K*_i_ values using the Cheng − Prusoff equation (Cheng and Prusoff 1973).

### Data analysis

Data shown are means ± SEM. Statistical significance was estimated by analysis of variance followed by Bonferroni´s t-test. A p-value < 0.05 was considered as significant.

### Drugs and materials

LSD was supplied as a stock solution from Merck, Germany. All other chemicals were of analytical grade. Demineralized water was used throughout the experiments. Stock solutions were freshly prepared daily.

## Results

1. **Left atrium from H**_**2**_**-TG**

**Fig. 2:**
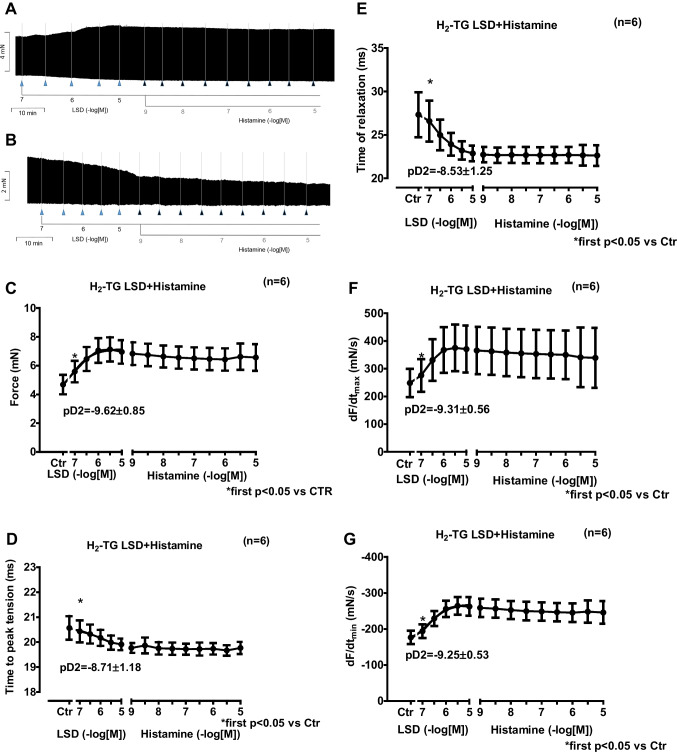
Original recording depicting the effect of LSD and additionally applied histamine on force of contraction in isolated electrically stimulated left atrial preparations from mice with cardiac overexpression of the human H_2_-histamine receptor (**A**) or littermate wild-type mice (WT, **B**). Arrows indicate drug application. Concentrations are given in negative decadic logarithms of lysergic acid diethyl amide (LSD) or histamine. Horizonal bars: ten minutes (10 min). Vertical bars force of contraction in milli Newton (mN). Force generated by LSD alone or additionally applied histamine (**C**) in milli Newton (mN) or time to peak tension in milliseconds (ms) (**D**), or time of relaxation (**E**), or rate of tension development (**F** in mN/ms), or rate of tension relaxation (**G** in mN/ms) in left atrial preparations from H_2_-TG. Ctr indicates pre-drug values. * indicates the first significant (*p* < 0.05) difference versus Ctr. Number in brackets indicates the number of experiments. The pD2 values for the effect of LSD are given

We first cumulatively applied LSD (0.1 µM to 10 µM). Subsequently, histamine was additionally and cumulatively applied. In left atrial preparations from H_2_-TG (Fig. 2A) but not from WT (Fig. 2B), LSD exhibited a time- and concentration-dependent positive inotropic effect that was not stimulated further by histamine (Fig. 2A). In WT, neither histamine nor LSD had a positive inotropic effect (Fig. 2B). These data are summarized with regard to the force of contraction for H_2_-TG (Fig. 2C). Moreover, in the same samples, LSD shortened the time to peak tension (Fig. 2D) and the time of relaxation Fig. 2E). Furthermore, LSD also augmented the absolute values of the rate of tension development (Fig. 2F) and the rate of relaxation (Fig. 2G). Subsequently applied histamine (1 nM to 10 µM) did not add to the effect of previously applied LSD.

2. **Right atrium from H**_**2**_**-TG**

**Fig. 3:**
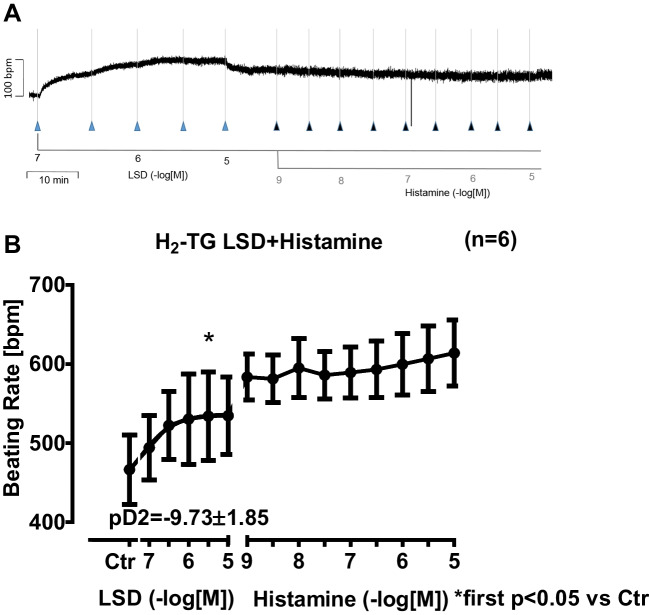
Original recording depicting the effect of LSD and additionally applied histamine on beating rate (in beats pro minute, bpm) in spontaneously beating isolated right atrial preparations from mice with cardiac overexpression of the human H_2_-histamine receptor (**A**). Concentration-dependent effects of LSD alone or in the additional presence of histamine in beats per minutes (bpm) (**B**). Ctr indicates pre-drug values. * indicates the first significant (*p* < 0.05) difference versus Ctr. Number in brackets indicates the number of experiments

Next, we were interested in right atrial function, under the same experimental conditions used in the left atrium. LSD time- and concentration-dependently increased the beating rate of right atrial preparations from H_2_-TG but not from WT. This is displayed in an original muscle strip in Fig. 3A (H_2_-TG). Summarized data for the beating rates can be found in Fig. 3B (H_2_-TG).

3. **Left atrium from 5-HT**_**4**_**-TG**

**Fig. 4:**
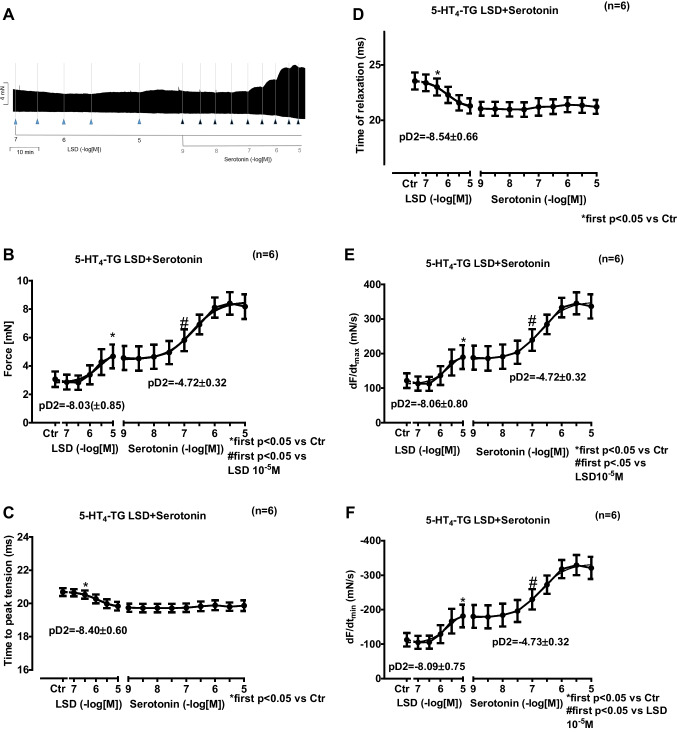
Original recording depicting the effect of LSD and additionally applied serotonin on force of contraction in isolated electrically stimulated left atrial preparations from mice with cardiac overexpression of the human 5-HT_4_-serotonin receptor (**A**). Arrows indicate drug application. Concentrations in negative decadic logarithms of lysergic acid diethyl amide (LSD) or serotonin. Horizonal bars: ten minutes (10 min). Vertical bars force of contraction in milli Newton (mN). Concentration-dependent effects of LSD alone or additionally applied serotonin on isometric force of contraction (**B**) in milli Newton (mN) or time to peak tension in milliseconds ms (**C**), or time of relaxation (**D**), or rate of tension development (**E** in mN/s), or rate of relaxation (**F** in mN/s) in left atrial preparations from 5-HT_4_-TG. Ctr indicates pre-drug values. * indicates the first significant (*p* < 0.05) difference versus Ctr. ^#^ indicates the first significant (*p* < 0.05) difference versus the highest concentration of LSD. Number in brackets indicates the number of experiments. Furthermore, the pD2 values are given for the effect of LSD and serotonin respectively

In separate studies, we first cumulatively applied LSD (0.1 µM to 10 µM). Subsequently, serotonin was cumulatively applied. In left atrial preparations from 5-HT_4_-TG, LSD exhibited a concentration- and time-dependent positive inotropic effect that was stimulated further by serotonin (Fig. 4A). These data are summarized in Fig. 4B with regard to force of contraction. Moreover, in the same samples, LSD shortened the time to peak tension (Fig. 4C) and the time of relaxation (Fig. 4D) in **5-HT**_**4**_**-TG**. Furthermore, LSD also augmented the absolute values of the rate of tension development (Fig. 4E) and the rate of relaxation (Fig. 4F). Subsequently applied serotonin (1 nM to 10 µM) added to the effect of previously applied LSD. Regarding the effect on the increase in force of contraction, we calculated the effect of LSD relative to that of LSD plus additionally applied serotonin, which was 29.37% ± 8.89%.

4. **Right atrium from 5-HT**_**4**_**-TG**

**Fig. 5:**
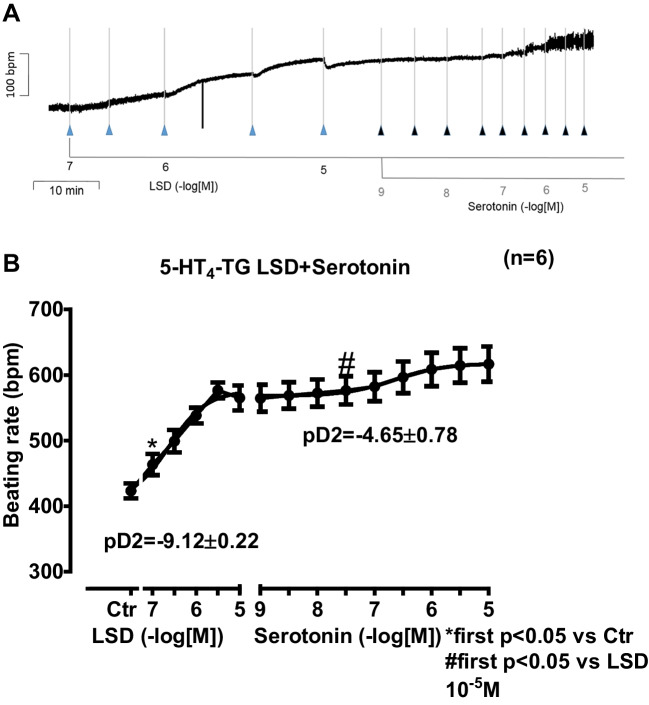
Original recording depicting the effect of LSD and subsequently applied serotonin on beating rate (in beats pro minute, bpm) in spontaneously beating isolated right atrial preparations from mice with cardiac overexpression of the human 5-HT_4_-serotonin receptor (**A**). Concentration-dependent effects of LSD alone or additionally applied serotonin (**B**) in beats per minutes (bpm) Ctr indicates pre-drug values. * indicates the first significant (*p* < 0.05) difference versus Ctr. Number in brackets indicates the number of experiments

Next, we were interested in right atrial function under the same experimental conditions as used in the left atrium. LSD time- and concentration-dependently increased the beating rate of right atrial preparations from 5-HT_4_-TG (original muscle strip in Fig. 5A). While LSD increased the beating rate in 5-HT_4_-TG, additionally applied serotonin did not stimulate the beating rate any further (Fig. 5A). Regarding the effect on the beating rate, we calculated the effect of LSD relative to the effect of LSD plus additionally applied serotonin, which was 76.69% ± 6.65%.

5. **Antagonists**

**Fig. 6:**
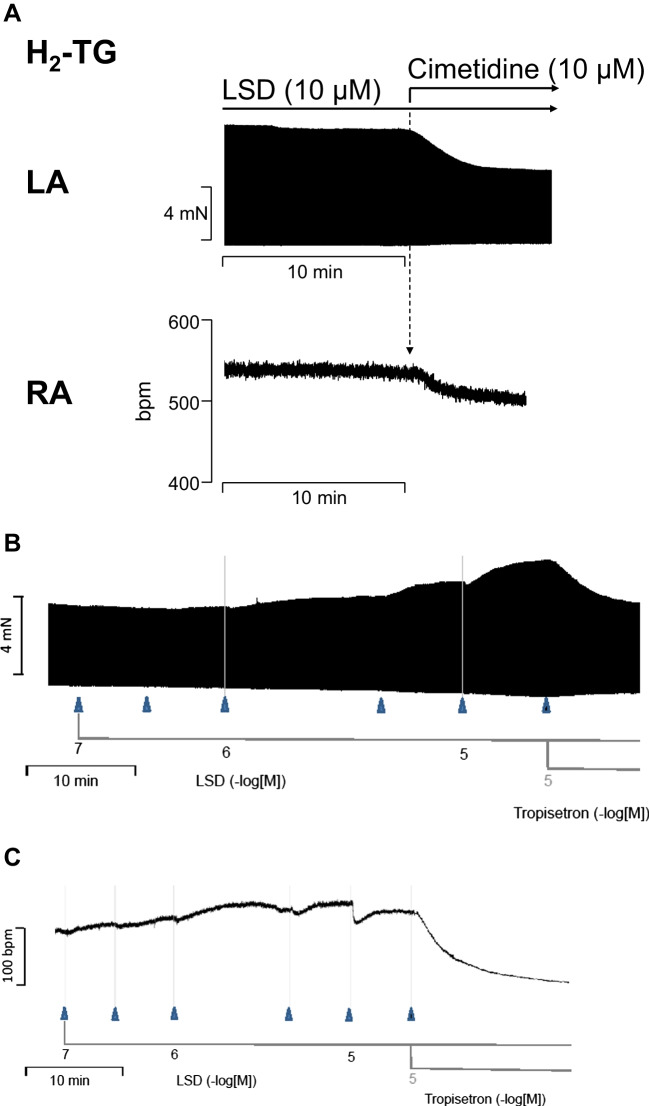
Original recordings: cimetidine antagonizes the positive inotropic effects of LSD (**A**, top) in left atrial preparations and the positive chronotropic effects of LSD in right atrial preparations from H2-TG (**A**, bottom). Tropisetron antagonizes the positive inotropic effects of LSD (**B**) in left atrial preparations and the positive chronotropic effect of LSD in right atrial preparations from 5-HT4-TG. Ordinates give force of contraction in milli Newton (mN) or indicate beats per minute (bpm). Horizontal bars indicate time scales in minutes (min). The arrows indicate the addition of drugs with the concentrations of drugs given in negative decadic logarithms

To confirm the role of the H_2_ receptor and the 5-HT_4_ serotonin receptor in transgenic mice, we used antagonists: cimetidine antagonized the positive inotropic effect or the positive chronotropic effect of LSD in H_2_-TG (original recording: Fig. 6A). Likewise, tropisetron antagonized the positive inotropic effect or the positive chronotropic effect of LSD in 5-HT_4_-TG (Fig. 6B, C). We chose to use tropisetron because it was the first antagonist used to define 5-HT4 receptors in the human atrium and had a pKb value of 6.7 in human atrium contraction studies (Kaumann et al. 1990).

6. **Langendorff perfused hearts**

Next, it was of interest to investigate ventricular effects of LSD. To this end, we used isolated retrogradely perfused hearts (Langendorff preparations), allowed to beat spontaneously. We recorded force of contraction from the apex and measured ventricular function under these conditions. We noted that 10 µM LSD increased force of contraction in hearts from H_2_-TG and, to a lesser extent, 5-HT_4_-TG, but not in hearts from WT animals(Table 1).

7. **Protein phosphorylation in mouse samples**

**Table 1:** Maximum effect of LSD (10 µM) on force of contraction in milli Newton (mN) and the rate of tension relaxation mN/seconds (mN/s) in isolated perfused hearts from H_2_-TG, 5-HT_4_-TG and WT. # indicate *p* < 0.05 versus pre-drug (10 µM LSD) value. *N* = number of animals

	WT	H_2_-TG	5-HT_4_-TG
*N* =	5	5	5
Basal force (mN)	12.6 ± 2.7	9.0 ± 1.8	11.6 ± 3.4
Force after LSD (mN)	14.9 ± 2.6	15.1 ± 2.6#	14.8 ± 3.5#
Basal rate of relaxation (mN/s)	176 ± 38.7	165 ± 33.3	215 ± 63.6
Rate of relaxation after LSD (mN/s)	186 ± 38.5	315 ± 55.6#	308 ± 85.6#

**Fig. 7:**
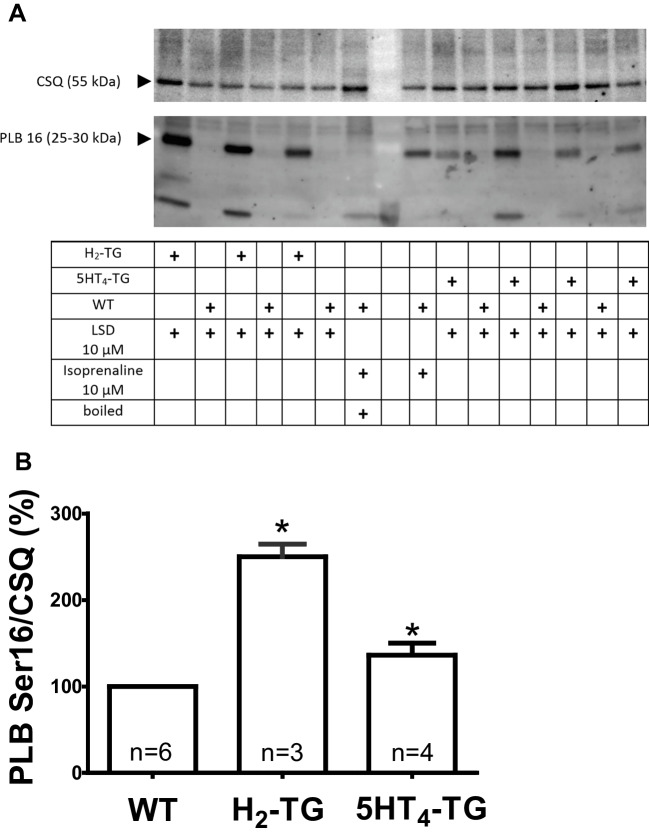
Photograph **A** of a Western blot. Left atrial contracting preparations from mice with cardiac overexpression of human H_2_-histamine receptors (H2-TG or human 5-HT_4_ receptors (5-HT_4_-TG) or wild type (WT) were stimulated with LSD or isoprenaline (positive control), freeze- clamped and homogenized. One sample of an isoprenaline-treated atrial preparation was boiled immediately before running the polyacrylamide gel. Samples were (molecular weight with arrows on the left) transferred to nitrocellulose. The upper part detects calsequestrin (CSQ; see Fig. 1) the lower part serine 16 phosphorylated phospholamban (PLB). Note the molecular weight reduction of the boiled sample which is characteristic of PLB. The gel was quantified **B** and the ratio of the signal for serine-16 phosphorlyated PLB and CSQ is plotted on the ordinate. Numbers in bars indicate the number of experiments. The ratio in WT samples was arbitrarily set as 100 percent

In freeze-clamped isolated atrial preparations, LSD increased the phosphorylation state of phospholamban at amino acid serine 16 in preparations from H_2_-TG and, to a lesser extent, 5-HT_4_-TG. Figure 7A displays a typical Western blot. Data are statistically analyzed in Fig. 7B.

8. **Effects in human atrium**

**Fig. 8:**
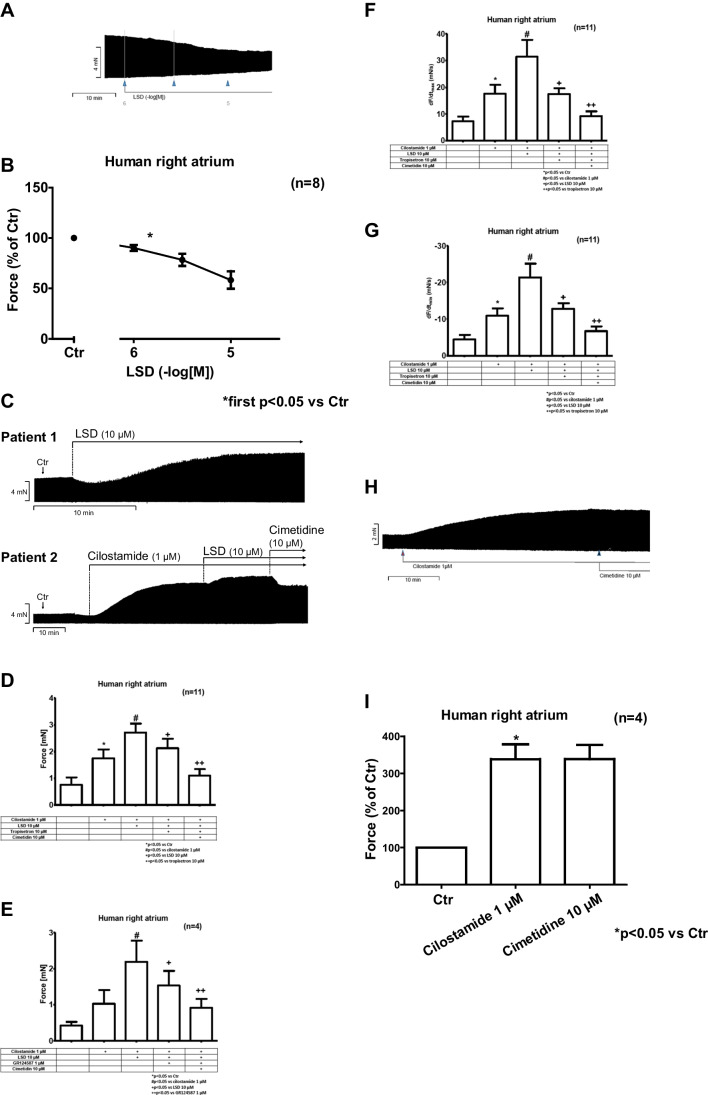
Original recording (**A**) of the effect of LSD without preincubation with Cilostamide on the force of contraction in isolated electrically stimulated right atrial preparations from patients undergoing bypass surgery. (**B**) Summarized relative effect of LSD without preincubation with Cilostamide on the force of contraction in human right atrial preparations(absolute mean force at Ctr 4.97 mN, after 10 µM LSD 2.89 mN). (**C**) Original recording depicting the effect of LSD alone (patient 1) or after pre-stimulation with 1 µM cilostamide and subsequently additionally applied cimetidine (patient 2) on force of contraction in human atrial preparations. (**D**) Summarized effects of subsequently applied cilostamide (1 µM), LSD (10 µM), tropisetron (10 µM) and cimetidine (10 µM) on the force of contraction of human atrial preparations. (**E**) Summarized effects of cilostamide (1 µM), LSD (10 µM), GR124587 (10 µM) and cimetidine (10 µM) on the force of contraction of human atrial preparations. (F, G) Summarized effects of cilostamide (1 µM), LSD (10 µM), tropisetron (10 µM) and cimetidine (10 µM) on the maximum rate of tension (**F**) and relaxation (**G**) development of contraction in human atrial preparations. (H, I) Original recording (**H**) and summarized relative data (**I**) showing the effect of 1 µM cilostamide and additionally applied 10 µM of cimetidine on the force of contraction in human atrial preparations(absolute mean force at Ctr 0.90 mN, after 1 µM cilostamide 3.05 mN, after 10 µM cimetidine 3.04 mN). Effects on force of contraction are given in mN or as % of pre-drug values respectively. Effects on maximum rates of tension or relaxation development are given in mN/s. Arrows indicate drug application. Horizonal bars: ten minutes (10 min). Ctr indicates pre-drug values. *p < 0.05 versus Ctr. Number in brackets indicates the number of experiments

In isolated human right atrial preparations, we first investigated the effect of LSD only. Usually LSD failed to show any positive inotropic effect (original recording in Fig. 8A, summarized data in Fig. 8B). Even a negative inotropic effect was apparent. Only in one preparation, LSD showed a positive inotropic effect without pre-incubation (Fig. 8C, patient 1). We had expected from the findings in in left atrial preparations from H_2_-TG and 5-HT_4_-TG that LSD alone would increase force of contraction. As that was usually not the case, in isolated human atrial preparations, we routinely measured the effect of LSD in the presence of 1 µM cilostamide. Cilostamide inhibits phosphodiesterase III which is the main phosphodiesterase in the human heart (e.g. von der Leyen et al. 1991). We hypothesized therefore that pre-stimulation with cilostamide would sensitize the human atrial preparations for positive inotropic effects of LSD. Initially, cilostamide on its own increased force of contraction, as expected from a PDE III-inhibitor in the human heart. Thereafter, additionally applied LSD raised force of contraction further. This positive inotropic effect of LSD was completely reversed by additionally applied cimetidine (Fig. 8 C, Patient 2) and partially reversible by additionally applied 10 µM tropisetron (Fig. 8D) or 1 µM GR125487 (Fig. 8E). We used here also GR125487 because it has a higher affinity to the 5-HT4-receptor than tropisetron (pKi-values of 10.1 and 6.8, respectively, Brattelid et al. 2004). Similarly, cilostamide and additionally applied LSD raised the absolute maximum rates of tension development (Fig. 8 F) and of relaxation development (Fig. 8 G), while additionally applied tropisetron or GR125487 and cimetidine reduced force of contraction elevated by LSD. One might thus be tempted to conclude that the effect of LSD in human atrial tissue might be mediated via both H_2_—and 5-HT_4_-receptors. Moreover, we also studied as a control the effects of tropisetron (10 µM) alone or GR124587 (1 µM) alone on force of contraction in human atrial preparations, which was not decreased significantly (to 97 ± 8.5% and 95 ± 9.3%, each *n* = 3, respectively). As a further control, we conducted experiments to determine whether the positive inotropic effect of cilostamide 1 µM itself might be reversed by subsequently applied 10 µM cimetidine, which it was not (original recording in Fig. 8H, summarized data in Fig. 8I).

9. **Radioligand binding studies**

**Fig. 9:**
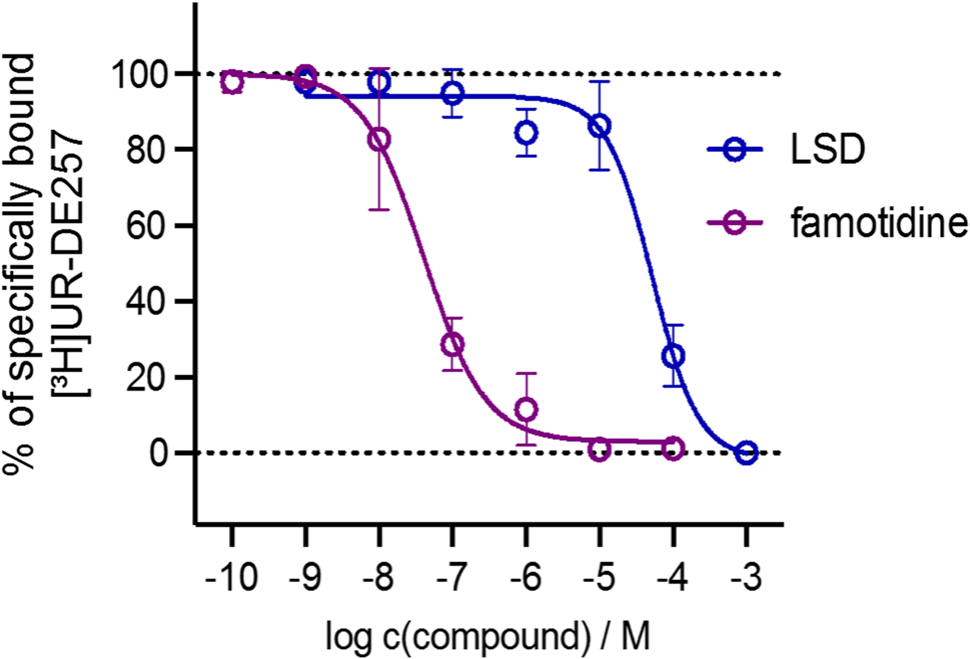
Displacement curve from a representative radioligand competition binding experiment performed with LSD, famotidine and [^3^H]DE257 (*K*_d_ = 66.9 nM, c = 40 nM, B_max_ = 11,122 dpm, corresponding to 990,000 receptor sites/cell) using HEK293-SP-FLAG-hH2R cells

In order to investigate the binding behavior of LSD we tested its affinity to the H_2_R in a radioligand binding assay using HEK cells in a recombinant expression system. Therefore, we used the selective H_2_R radioligand [^3^H]UR-DE257 (*N*-[6-(3,4-dioxo-2-{3-[3-(piperidin-1-yl-methyl)phenoxy]propylamino}cyclobut-1-enylamino)hexyl]-(2,3-^3^H_2_)propionic amide, *K*_d_ = 66.9 nM, c = 40 nM, B_max_ = 11,122 dpm, corresponding to 990,000 receptor sites/cell), that is useful for the identification and pharmacological characterization of H_2_R ligands (Baumeister et al. 2015). LSD showed binding at the human H_2_ receptor (p*K*_i_ = 4.49 ± 0.09, slope = 1.31 ± 0.11, *n* = 3, Fig. 9), which is depicted in Fig. 9 in direct comparison to reference compound famotidine (p*K*_i_ = 7.63). Unspecific binding was detected using famotidine (10 µM).

## Discussion

The first evidence for LSD action at H_2_ receptors was found in membranes from guinea pig brains, where LSD increased the activity of adenylate cyclase in a cimetidine sensitive manner, and thus proved to be H_2_ receptor mediated (Green et al. 1978). Indeed, our data in transgenic animals indicate that LSD is an agonist at human H_2_ receptors as the effects of a high concentration of LSD reached a plateau, indicating full receptor saturation. Under these conditions, exogenous histamine was ineffective. The evidence that LSD exerted these actions via H_2_ receptors is several fold. Firstly, LSD was only active in H_2_-TG samples and not in WT samples. Secondly, the effect of LSD on force and beating rate in H_2_-TG samples could be antagonized by cimetidine, known as a selective antagonist at H_2_ receptors. Thirdly, LSD led to an increase in phosphorylation of PLB (Fig. 1), a pathway expected for H_2_ receptor agonists (Gergs et al. 2019). Fourthly, in transfected cells, LSD could bind to H_2_ receptors. Moreover, our findings are in line with previous animal studies where LSD could increase force or/and beating rate in rabbit or guinea-pig cardiac muscle strips (Angus and Black 1980). These effects were blocked by cimetidine (Angus and Black 1980).

We proved that stimulation of H_2_ receptors is clinically relevant in humans when the inotropic effect of LSD in isolated human heart samples was blocked by cimetidine, thus demonstrating a H_2_ receptor mediated response. Moreover, we would argue the fact that we usually required phosphodiesterase III inhibition by cilostamide argues that also in the human heart H_2_ receptors couple LSD to force generation. There are data that H_2_ stimulation in human heart samples leads to cAMP increases and activation of cAMP dependent protein kinase (Fig. 1, review: Neumann et al. 2021a, b, c). H_2_ activation is expected to activate PKA, which only phosphorylates PLB on serine 16 (Simmerman et al. 1986). This augmented phosphorylation of PLB explains at least in part the relaxant effects and inotropic effects of LSD in the human atrium.

LSD has a role as a recreational drug or drug of abuse (De Gregorio et al. 2016). LSD is used for these purposes probably because LSD is very potent agonist a 5-HT_2A_ receptors, where binding is thought to lead to hallucinations that some users crave for. In conjunction, it is almost certain that more than one receptor is involved in the central nervous system actions of LSD because it has long known that LSD has a high affinity for many G-protein coupled receptors (Roth et al. 2002). For instance, LSD binds with high affinity to all five dopamine receptors and most serotonin receptors (Roth et al. 2002). Hence, the signal transduction of LSD is complex (review: Olson 2022). However, LSD binding to 5-HT_4_ receptors appears to be unreported.

PLB is only expressed in cardiomyocytes and not in non-cardiomyocytes. The fact that we measured an increase in PLB phosphorylation in atrial samples is strongly indicative that the H_2_ receptors are present and functional in cardiomyocytes. We also used the alpha myosin promotor, which drives expression in the heart only in cardiomyocytes (Subramaniam et al. 1993).

## Clinical relevance

Our data have clinical applications. Firstly, intoxications with LSD have been well reported. Intoxication with LSD were accompanied with tachycardia by 40% of recreational users, demonstrating that cardiac side effects of LSD are clinically relevant (Leonard et al. 2018, Passie et al. 2008).

Should these intoxications be accompanied by cardiac arrhythmias, our data suggest that it might be worthwhile to test cimetidine as an antidote. Cimetidine is an approved drug and, when given intravenously, should block any effects of LSD on the cardiac H2-R that may have directly caused an arrhythmia. Alternatively, one could also use the hH2-R antagonist ranitidine. Ranitidine is more potent than cimetidine and is available in an injectable drug formulation. Likewise, it is conceivable to try to terminate cardiac arrhythmias in LSD overdosing by additionally applying tropisetron intravenously.

As well as intoxication treatment options, our data might supply evidence for LSD to be used as a therapeutic drug. Recently, efforts have been renewed to treat psychiatric patients with LSD (Gasser et al. 2015). Indeed, there are currently 148 studies on clinicaltrials.gov for LSD. Our data will allow calculations for safe plasma concentrations of LSD, to act on high affinity brain receptors but not on cardiac H_2_ receptors. We also show that in addition, 5-HT_4_ receptors might similarly affect the human heart. Reported therapeutic plasma levels after taking 100 µg perorally LSD is 1.3 ng/ml (about 4 nM, Dolder et al. 2018), and for recreational purposes about fivefold higher doses were reported, leading to 20 nM plasma concentrations of LSD (Dolder et al. 2018). However, these plasma concentrations might be higher if the metabolism of LSD is impaired either by additionally applied drugs or when (for genetic reasons) patients exhibit low metabolism of LSD (Luethi et al. 2019, Table 2).

**Table 2:** Reported plasma levels of LSD in humans

	Plasma levels of LSD in humans	Reference
Therapeutic LSD dose	4 nM^1^, 5.5 nM^2^, 5.3 mM^3^	^1^Dolder et al. 2018 ^2^Holze et al. 2020 ^3^Holze et al. 2019
Recreational dose	1.1 nM^2^, 20 nM^1^	^1^Dolder et al. 2018 ^2^Martin et al. 2012
Overdose	20 nM (calculated)^1^, 5.9 nM ^2^	^1^Mathew 1968 ^2^McCarron et al. 1990

In order to compare our concentrations with those LSD reported in clinical situations, the plasma levels were listed in some typical studies for therapeutic and toxic peak levels of LSD. Note the overlap

## Limitations

We have not had the opportunity to study human ventricular samples in our contraction study due to lack of access to that tissue. Hence, we can only extrapolate from our Langendorff data in H_2_-TG that LSD will also have effects on the human ventricle. Moreover, there is a negative inotropic effect of LSD in WT in Fig. 2B. This cannot result from stimulation of endogenous mouse H_2_-receptors because histamine itself does not reduce force of contraction in isolated left atrial preparations from wild-type mice (Gergs et al. 2019). In line with negative inotropic effect in left atrium from WT, we report in Fig. 8A a similar cardioinhibitory effect of LSD alone (in the absence of cilostamide) in human atrial preparations. It is generally accepted that histamine only increase and does not decrease force of contraction in the isolated human atrium (e.g. Baumann et al. 1981). We would speculate, the another, presently unknown receptors, to which LSD binds, probably underlies this effect. The actual mechanism(s) need(s) of the negative inotropic effect of LSD remain(s) to be elucidated.

Moreover, we cannot explain, why in the whole set of patients we studied, only in one patient we measured a pronounced positive inotropic effect of LSD alone. We could speculate that this patient has genetically a higher expression of the 5-HT4-receptor in the heart than other patients which would explain this finding.

In conclusion, LSD can increase force of contraction via stimulation of human H_2_ histamine receptors and human 5-HT_4_ receptors, in isolated atria from H_2_-TG and 5-HT_4_-TG, but also in the isolated human atrium.

## Data Availability

The data of this study are available from the corresponding author upon reasonable request.
